# The effect of instrumental and environmental factors on the thermal regulation of the temperature of incubation – IFCC Document (1986)

**DOI:** 10.1155/S146392468700018X

**Published:** 1987

**Authors:** C. A. Burtis

**Affiliations:** Chemical Technology Division Oak Ridge National Laboratory PO Box X Bldg. 4500N Oak Ridge Tennessee 37831-6194 USA


**Journal of Automatic Chemistry**/*Journal of Clinical Laboratory Automation*, **Vol. 9, No. 2 (April–June 1987), pp. 82–86**

### The effect of instrumental and environmental factors on the thermal regulation of the temperature of incubation — IFCC Document (1986)

*Prepared for, and on behalf of, the International Federation of Clinical Chemistry's Expert Panel on Instrumentation by:*

**C. A. Burtis**

*Chemical Technology Division, Oak Ridge National Laboratory, PO Box X, Bldg. 4500N, Oak Ridge, Tennessee 37831–6194, USA*

### Preface

In 1982 the International Federation of Clinical Chemistry's (IFCC), Expert Panel on Instrumentation, issued a statement recommending that 37 °C be the preferred temperature of incubation for routine methods in clinical chemistry.

Subsequently, the IFCC Scientific Committee formed a joint working party, consisting of Dr R Rej, the Chairman of the Expert Panel on Enzymes (EPE), and Dr C. A. Burtis, a member of the Expert Panel on Instrumentation (EPI), to prepare a report on incubation temperature. After initial discussions, it became apparent that basically there were two fundamental aspects of the temperature question: (1) the thermodynamic effect on the analytical process in question; and (2) the thermal regulating capabilities of an instrument in the environment in which it is expected to operate. With regard to the latter, to achieve and maintain temperature regulation in a reaction cuvette, the opposing actions of processing that add and remove heat to its contents must be balanced. To achieve this balance within an acceptable time frame, heat is first generated and supplied by a regulated heat source. Heat can then be removed either passively by allowing it to dissipate into the environment or actively by some type of regulated device. Since the ambient temperature of the environment in which an instrument is operated has a direct and significant effect on the effectiveness of passive cooling, regulated cooling is needed when the temperature differential ($\Delta T$) between the ambient temperature and the set point temperature is less than 5 °C. Since it is often difficult to maintain a $\Delta T$ of 5° at a set point of 30 °C, 37 °C was recommended by the EPI in 1982. However, in some countries during the hot, summer seasons, the prerequisite $\Delta T$ of 5 °C is difficult to maintain even at 37 °C.

Expert Panel (Scientific Committee Analytical Section, IFCC) members are: T. D. Geary (Australia), *Chairman*, N. Alpert (USA), P. Bonini (Italy), C. A. Burtis (USA), and T. Sasaki (Japan).

Comments on the document, including comments from the viewpoint of languages other than English, are encouraged and should be sent to Dr Burtis at the Oak Ridge National Laboratory.

Since modern instruments are designed to operate world-wide in a variety of environments, *the EPI recommends that future instruments be designed to provide both regulated heating and cooling capabilities*. Improved temperature control should result and the effect of ambient temperature will be minimized.

The following document has been prepared in support of this recommendation by Dr Burtis on behalf of the EPI.

### Introduction

To ensure comparable clinical results, considerable effort has been, and will continue to be, expended on the development of a standardized measurement system for use by clinical laboratories world-wide. As a consequence of these standardization efforts, numerous reference materials and methods are now available which have received consensus acceptance by a variety of national and international organizations.

One area of standardization which has proven to be controversial and for which consensus has not been obtained is that of selection of a single reaction temperature at which all clinical analyses will be performed. During the past several years, various temperatures have been suggested and recommended [1–8]; however, the continuing debate has narrowed the choice down to either 30 °C or 37 °C. Recently, on behalf of the Expert Panels on Enzymes (EPE) and Instrumentation (EPI) of the International Federation of Clinical Chemistry (IFCC), an international survey on the topic of temperature selection was conducted and the results published [9]. As would be expected from the history of the temperature controversy, this document lists reasonable arguments for selection of either temperature. Clearly, the question of temperature selection is a difficult problem and one for which an indisputable selection cannot be made.

There are two major components of the temperature question, which can be categorized as analytical and instrumental. Analytically, temperature is one of the variables which an analyst can use to offset the kinetic and steady-state thermodynamics of processes and phenomena such as reaction rates, antigen-antibody binding and chemical complexes whose absorbance spectra is temperature dependent [10 and 11]. Instrumentally, the selection of a specific set point temperature as a set point about which the temperature of a reaction is to be maintained has a direct effect on the design and cost of an analytical instrument and its ability to achieve and maintain thermal regulation in the environment in which it will be operated. The purpose of this document is to discuss the various instrumental factors which influence thermal regulation and how they interact with those of the environment in which an instrument is to be operated.

### Temperature regulation

The regulation of the temperature of a liquid in a reaction cuvette at a specific set point is a process in which the heat flux within the chamber is maintained and regulated for a specific period of time (for example a steady-state is achieved). In practice, this entails the balancing of the opposing actions of heat input and heat loss via the several processes which add and remove heat to and from the system [12]. A block diagram of a generalized temperature control system is shown in figure 1.

The function of any automatic control system is to monitor a process variable (i.e. temperature of the liquid) and to alter controlled parameters (i.e. heat fluxes) via a control device to form a closed loop of action and response which results in the desired system behavior [13]. In the generalized case shown in figure 1, there is a feedback of information from the measured temperature of the process ($T_m$) to a controller which then regulates temperature at a preset value ($T_{sp}$) while minimizing excursions. Such a process is termed 'feedback control'.

### Temperature measurement

The first step in the thermal regulation of a process is the measurement of the temperature in the reaction cuvette. This measurement is made by some type of a temperature sensing device (usually a calibrated thermister, thermocouple, thermometer, or thermochromic solution), which is placed in or near to the reaction cuvette. Measurements are generally based on calibrations which compensate for effects resulting from the placement of the temperature sensing device.

### Heat addition

When the measured temperature of the reaction cuvette is less than the set point ($T_m < T_{sp}$) heat ($q$) is required and is added to the chamber until a thermal steady-state is obtained which results in $T_m = T_{sp}$. The total heat added is a combination of the heat generated from a regulated heat source (active heating) and that supplied from unregulated sources (passive heating).

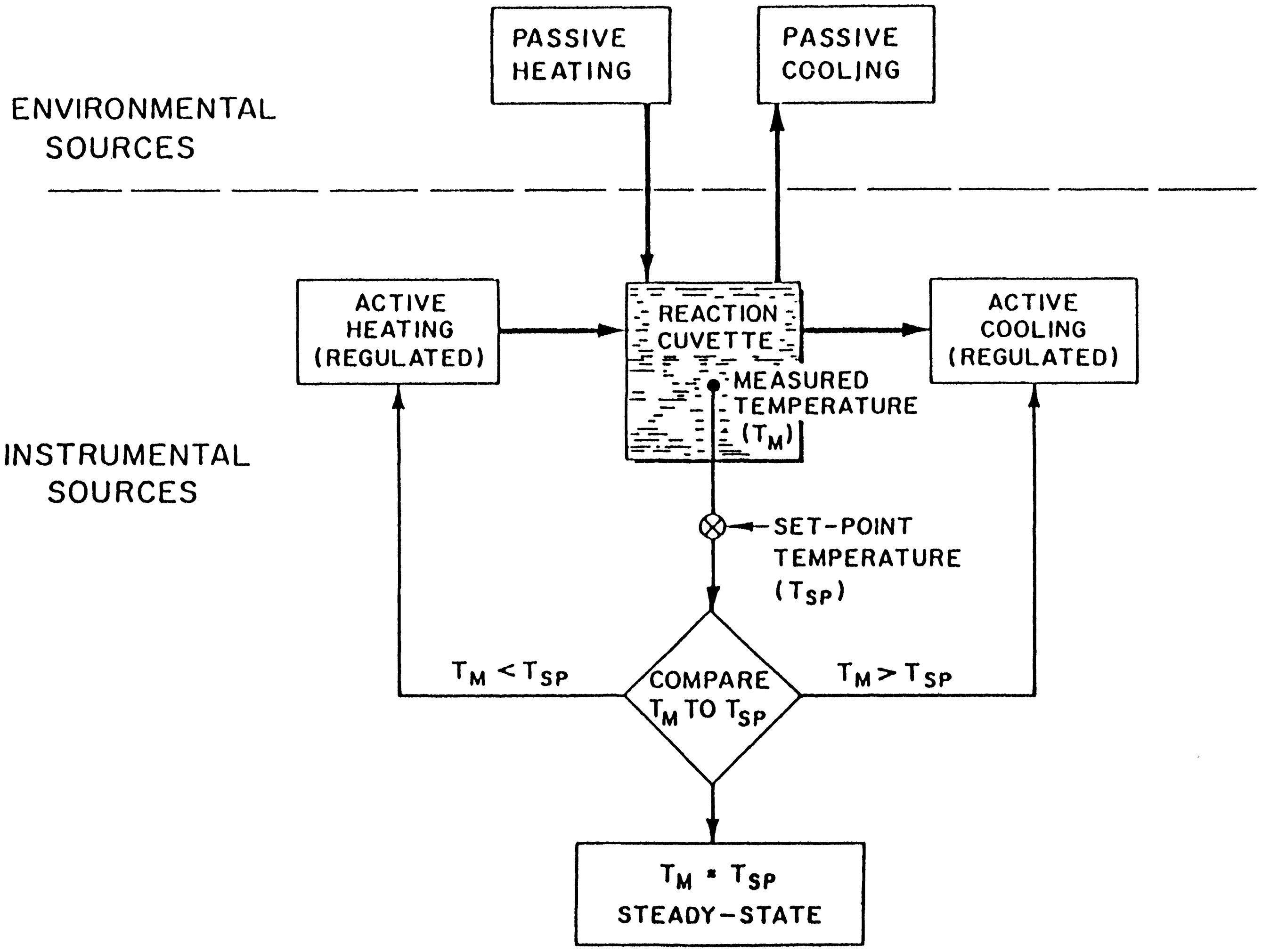

*Figure 1. Schematic representation of a thermal regulation system and the effect of environmental heat sources on it.*

*Regulated heat source*

A regulated heat source actively generates a specific quantity of heat in a time frame consistent with the overall time requirements of the system. In a simple control system, the quantity of heat provided is a function of the temperature difference ($\Delta T$) between $T_m$ and $T_{sp}$ and the quantity of heat supplied from the unregulated, external sources. For a thermal regulating system to function properly, the regulated heat source should provide the predominant percentage of the total heat input.

*Unregulated heat sources*

The ambient heat contained in the environment is one of the two major sources of passive or unregulated heat. When the ambient temperature approaches that of the set point and the resulting thermal differential becomes less than 5 °C, the passive heat supplied from the environment becomes the major component of the total heat input and, since it is unregulated, control of the process is subsequently lost.

The second source of unregulated quantities of heat is the heat produced from the routine operation of the system. When this system is a large, multicomponent device, then considerable quantities of heat are generated during its routine operation. When the environment in which the system operates is controlled, then this endogenous heat can be dissipated into the environment which functions as a high capacity heat sink. When the environment is not controlled and the ambient temperature approaches that of the set point, then the heat capacity of this heat sink becomes limiting and this endogenous heat can become a significant proportion of the total heat input. Thermal steady-state is then difficult to maintain.

### Heat transfer

As a specific quantity of heat is generated, it is transferred to the reaction cuvette at a given rate. Its conduction is a function of the cross-sectional area of the cuvette, the temperature gradient across it, and the thermal conductivity of the conducting medium [14]. There are three fundamental types of heat transfer: conduction, convection, and radiation. All three may occur at the same time but in any given system one is designed to predominate. Factors which influence the effectiveness of transfer by conduction are the heat conductivities and capacities of the materials used to fabricate the cuvette and also its contents. In general, the heat transfer into and out of the cuvette will be faster through its walls than through its liquid contents since the thermal conductivity of solids is approximately 100 times greater than that of liquids [14].

### Heat loss

In order to maintain a constant heat flux through the reaction cuvette, its thermal regulating system must have the capability not only to generate and supply heat but also to remove excess heat. Heat can be actively removed by a regulated heat sink or passively lost through unregulated heat sinks.

*Regulated heat sinks*

When $T_m > T_{sp}$, a quantity of heat has to be removed from the reaction cuvette in order to achieve thermal steady-state with $T_m = T_{sp}$. With a regulated heat sink, specific quantities of heat can be removed at a constant and predictable rate. In general, a thermal regulation system that incorporates a regulated heat sink into its design will cost more than those utilizing unregulated heating sinks for heat removal. However, such a system is capable of providing thermal regulation over a wider range of ambient and set point temperatures than systems which use unregulated heat sinks for cooling purposes.

*Unregulated heat sinks*

Systems which utilize unregulated heat sinks are designed to lose heat by allowing it to passively dissipate into the ambient environment. As a rule of thumb, it is commonly assumed that for such systems to function, the ambient temperature of the environment in which they operate has to be maintained (i.e. air-conditioned) at least 5 °C below that of the set point temperature. In addition, cooling fans are also often included in such systems to hasten heat transfer and dissipation and to remove moisture, thereby minimizing condensation.

In an uncontrolled environment, the ambient temperature can approach, and even exceed, the set point temperature. In this situation, passive dissipation of heat to the ambient environment does not occur.

### Discussion

In the previous sections, the individual components that constitute or affect the thermal regulation on an instrument reaction cuvette(s) were discussed. Using this information and the schematic shown in figure 1, the pertinent factors that will influence the selection of either 30 °C or 37 °C as the set point temperature can be objectively discussed.

*Availability of an international practical temperature scale standard*

The accuracy of a temperature measurement depends on its calibration and traceability to a reference point on the International Practical Temperature Scale (IPTS). Reference points having relevance to the clinical laboratory are the triple and boiling points of water. Recently, the melting points of gallium [15] at 29 772 °C and rubidium [16] at 39 30 °C have been proposed as additions to the IPTS and are available as certified reference materials from the National Bureau of Standards (NBS).

The availability of a reference standard that is traceable to the IPTS has no direct effect on an instrument's thermal regulating capabilities. However, a thermal regulating system which can be calibrated and traced to accurate temperature measurements would ensure its credibility and transferability between laboratories. Thus, any temperature selected as the set point should be traceable to the IPTS. The melting points of gallium and rubidium are close to the candidate temperatures of 30 °C

and 37 °C. Thus, a traceable and transferable accuracy base can be ensured for both temperatures.

*Environmental conditions*

The ambient environment in which it will be operated is one of the most important factors that is considered in the design and development of an analytical instrument. In addition, the interrelationship between the ambient environment and the thermal regulating capabilities of an instrument is a very important point to consider in selecting a set point temperature. As discussed earlier, the thermal regulation of a reaction cuvette and its contents entails the dynamic balancing of the opposing actions of heat input and loss. For this process to occur in a predictable and reproducible manner, heat fluxes must be controlled (figure 1) where possible and heat contributions from unregulated sources must be minimized. In practice, this latter situation is ensured by controlling the ambient temperature of the environment by air conditioning or localized cooling within the instrument. As a rule of thumb, when the ambient temperature is maintained at least 5 °C below that of the set point, the regulated heat source will provide most of the total heat input. In addition, when the thermal differential ($\triangle T$) between the ambient and set point temperatures is 5 °C or greater, than the ambient environment can also serve as a large, high-capacity heat sink into which excess heat can be transferred and passively dissipated. In fact, many instruments are purposely designed to use the ambient environment as the heat sink component of their thermal regulation system.

Thermal regulation systems begin to have problems functioning when the ambient temperature begins to approach that of the set point and the resultant thermal differential becomes less than the prerequisite 5 °C. Under this condition, the heat supplied from the unregulated source becomes the major component of the total heat input and, since it is unregulated, control of the process is subsequently lost. In addition, transfer and dissipation of heat into the ambient environment becomes limiting thereby precluding its use as a heat sink. Obviously, under this condition, instruments which have been designed to utilize the ambient environment as a component of their thermal regulation system would lose control of both the heat input and loss-regulating functions of their system.

With a design specification that the $\Delta T$ between the ambient and set point temperatures must be at least 5 °C or greater, then selection of a specific temperature as a set point places certain requirements on the environment in which an instrument will be operated. For example, at a set point of 30 °C, the ambient temperature must be maintained at 25 °C or less. Likewise, at 37 °C, the ambient temperature should be less than 32 °C. In an air-conditioned laboratory, the ambient temperature is maintained in a range of approximately 20–23 °C. Obviously, in such environments, either 30 or 37 °C could be selected and an acceptable $\Delta T$ maintained. However, many laboratories are not air conditioned and when they are located in areas that have warm to hot climates, the summer temperature can easily range in the upper 20s to low 30s. In fact, even air-conditioned laboratories located in such climates often have problems maintaining temperatures in the low 20s. Consequently, thermal regulation is difficult when 30 °C has been selected as the set point since a $\Delta T$ of at least 5 °C cannot be maintained. Even at a set point of 37 °C, problems may occur on extremely hot days.

To compensate for situations in which the ambient temperature approaches that of the set point, certain options are available to an instrument designer. One, the laboratory can be air-conditioned either centrally throughout the entire building or by use of a portable window air-conditioning unit to cool an individual room. However, in some situations, neither is feasible. A second option is to include a regulated heat sink into the design of the instrument. This approach has been termed 'active' cooling and requires the addition of a cooling unit into the instrument and a means of removing moisture before condensation can occur. Examples of such units are circulating water coolers, air coolers, air conditioners, or devices based on the 'Peltier' effect [17]. These latter devices can also be used to supply as well as remove heat.

*Mode of operation*

Whether an instrument operates in a manual or automatic mode has an impact on the selection on the set point temperature. One of the initial steps in the operation of a thermal regulation system is to first achieve thermal steady-state by adjusting heat fluxes so that the temperature of the contents of the reaction cuvette is maintained at the set point ($T_m = T_{sp}$). Then, the system maintains temperature control by adding or removing heat as necessary.

In an automated instrument, the thermal regulation system can be designed to achieve and maintain a desired temperature in a time frame that is consistent with its overall design criteria. In a manual instrument, the problem often encountered is the initial adjustment of the temperature of the reaction cuvette. Manual instruments often utilize a thermal regulation system which can only add heat — with excess heat being removed only by passive dissipation. In addition, the heat generating capacity and heat transfer characteristics of manual instruments often result in thermally sluggish systems (i.e. there is a long time lag between a control action and a system response). To compensate for such behavior characteristics, the temperature of the cuvette and its contents can be adjusted by a preheating step. The degree of adjustment required depends on the ambient temperature and the set point. If the ambient room temperature is, for example, 22 °, then the temperature of the cuvette has to be adjusted by either 8 ° or 15 °, depending on whether 30 °C or 37 °C is selected as the set point.

Usually, a preheating step is accomplished by placing the cuvette in an external thermostatted environment after which it is then replaced into the instrument. Studies have shown that even during the short time interval between removing the cuvette from the thermostatted environment, drying it, if necessary, and placing it in the thermally controlled compartment of the instrument, a drift in temperature occurs. The magnitude of the drift depends on the difference between the ambient and set point temperatures; it is greater at set point 37 °C than at 30 °C. Consequently, manual instruments are often operated at a set point temperature of 30 °C. Since the IFCC enzyme reference methods have been developed by the EPE using manual instruments, they have selected 30 °C as their recommended temperature.

*Instrumental costs*

A thermal regulating system (figure 1) involving regulated heating and cooling and strategically placed thermal sensors will provide the quickest and most tightly-controlled system to reach and maintain the temperature around a given set point. If properly designed, such a system would provide acceptable performance independent of the ambient temperature and the temperature selected as a set point. The primary disadvantage of such an instrument is cost since it would be more expensive to design and incorporate an active cooling component into its thermal regulation system. However, the resulting instrument would have the capability to operate in environments with a wider range of ambient temperatures irregardless of what specific temperature has been selected as a set point.

### References

1. Bergmeyer, H. U., Standardization of the reaction temperature for the determination of enzyme activity. *Z. Klin. Chem. Klin. Biochem.*, **11** (1973), 39–45.
2. Kieding, R., Horder, M., Gerhardt, W. *et al.*, Recommended methods for the determination of four enzymes in blood. *Scandinavian Journal of Clinical Laboratory Investigation*, **33** (1974), 291–306.
3. German Society of Clinical Chemistry, Recommendations for a uniform temperature for the estimation of enzyme activities in clinical chemistry. *Z. Klin. Chim. Klin. Biochem.*, **9** (1971), 464.
4. Société Française de Biologie Clinique, Standardisation de la temperature utilisee pour la measure des activities enzymatiques courantes en chimie clinique. *Ann. Biol. Clin.*, **33** (1975), 51.
5. Bowers, G. N. Jr., Bergmeyer, H. U., Horder, M. and Moss, D. W., IFCC methods for the measurement of catalytic concentration enzymes. Part 1. General considerations concerning the determination of the catalytic concentration of an enzyme in the blood serum of man. *Clinica Chimica Acta*, **98** (1979), 163F–174F, and *Journal of Clinical Chemistry and Biochemistry*, **18** (1980), 89–95.
6. Bretaudiere, J. P., The optimal reaction temperature for enzyme activity measurements: 30 °C, in *Proceedings of the Second International Symposium on Clinical Enzymology*, edited by N. W. Tietz, A. Weinstock, and D. O. Rodgerson (AACC Press, Washington, D.C., 1976), pp. 303–309.
7. Tietz, N. W., Batsakis, J., Bayse, D. *et al.*, Guidelines for photometric instruments for measuring enzyme reaction rates. *Clinical Chemistry*, **23** (1977), 2160–2162.
8. Statland, B. E., Enzyme assay conditions: choice of standard reaction temperature for assaying activity values of enzyme in human sera (Document C-10, National Committee for Clinical Laboratory Standards, Villanova, Pennsylvania, 1979).
9. Haeckel, R., Horder, M. and Zender, R., Report on the survey opinions concerning preferred incubation temperatures for measurement of enzymes (and possibly other components) in clinical chemistry. *Journal of Clinical Chemistry and Clinical Biochemistry*, **20** (1982), 947–958; and *Clinica Chemica Acta*, **129** (1983), 245F–249F.
10. Burtis, C. A., Seibert, L. E., Baird, M. A. and Sampson, E. J., Temperature dependence of the absorbance of alkaline solutions of 4-nitrophenyl phosphate. A potential source of error in the measurement of alkaline phosphatase activity. *Clinical Chemistry*, **23** (1977), 1541–1547.
11. Spierto, F. W., MacNeil, M. L. and Burtis, C. A., The effect of thermochromism on the measurement of creatinine. *Clinical Biochemistry*, **12** (1979), 18–24.
12. Chodosh, D. F., Levinson, S. H., Weber, J. L., Kamholz, K. and Berkoff, C. E., Automated chemical synthesis. Part 3: Temperature control systems. *Journal of Automatic Chemistry*, **5** (1983), 103–107.
13. Coughanowr, D. R. and Koppel, L. B. *Process Systems Analysis and Control* (McGraw-Hill Book Company, New York, (1965), pp. 101–110.
14. Bennett, C. O. and Myers, J. E., *Momentum, Heat and Mass Transfer* McGraw-Hill Book Company, Inc., New York, (1962), pp. 242–247.
15. Mangum, B. W., The gallium meeting point standard. Its role in our temperature measurement system. *Clinical Chemistry*, **23** (1977), 711–718.
16. Standard Reference Material 1969: *Rubidium Triple-Point Standard* (National Bureau of Standards, Washington D.C., 1984).
17. Hagelauer, U., Arnaudov, K. and Faust, U., Zur Problematik des Temperierens in der Enzymanalytic, *DT. Ges. f. Klin Chemie*, **5** (1984).

**FOOD ADDITIVES AND CONTAMINANTS**

The latest issue of this international quarterly includes the following papers, which may be of interest to *JAC* readers:

Collaborative evaluation of a procedure for the determination of *N*-nitroso compounds as a group.

Identification of substituted hydroxypyridines and hydroxypyrazines in caramel food colourings.

Nickel in foods and the diet.

*Sample copies from Taylor & Francis, Rankine Road, Basingstoke, Hampshire RG24 0PR, UK.*

